# Effect of femoral head necrosis cystic area on femoral head collapse and stress distribution in femoral head: A clinical and finite element study

**DOI:** 10.1515/med-2022-0506

**Published:** 2022-07-13

**Authors:** Zhaoming Zhang, Tianye Lin, Yuan Zhong, Wenting Song, Peng Yang, Ding Wang, Fan Yang, Qingwen Zhang, Qiushi Wei, Wei He

**Affiliations:** Department of Orthopedics, The First Clinical Medical College of Guangzhou University of Chinese Medicine, Guangzhou, Guangdong 510405, China; Department of Orthopedics, Affiliated Foshan Hospital, Guangzhou University of Traditional Chinese Medicine, Foshan 528000, China; Department of Orthopedics, Guangdong Research Institute for Orthopedic & Traumatology of Chinese Medicine, Guangzhou, Guangdong 510240, China; Department of Joint Orthopaedic, The Third Affiliated Hospital, Guangzhou University of Chinese Medicine, Guangzhou, Guangdong 510240, China

**Keywords:** osteonecrosis of the femoral head, cystic area, collapse, mechanism, finite element

## Abstract

The purpose of this study was to investigate the effect of cystic areas of osteonecrosis of the femoral head (ONFH) on stress distribution and disease progression in the femoral head. A total of 85 patients (106 hips) diagnosed with Association Research Circulation Osseous stage II non-traumatic and non-surgical treatment were retrospectively analyzed. The presence of cystic areas and diameter of cystic areas were compared between the two groups. In addition, five spherical cystic areas of different diameters were constructed and the maximum stress was observed. There was a difference between the two groups in whether cystic areas appeared in the femoral head, with 49.1% in the collapse group showing cystic areas, which was significantly higher than that in the non-collapse group (18.4%) (*P* < 0.05). In addition, the diameter of the cystic areas was significantly larger in the collapsed group than in the non-collapsed group (*P* < 0.05). The maximum and mean von Mises stress value around the necrotic area and around the cystic area of the femoral head increased with the increase of the cystic diameter. Stress concentration areas can be generated around the cystic areas. The presence and increased diameter of the cystic areas accelerates the collapse of the ONFH femoral head.

## 1 Introduction

Osteonecrosis of the femoral head (ONFH) is a disease in which the femoral head is interrupted or damaged, causing the death of osteocytes and bone marrow components, and subsequent repair leads to collapse of the femoral head, and the specific pathogenesis has not been fully clarified [1,2]. Changes such as crescent sign, cystic areas, and articular surface collapse occur radiologically during the progression of ONFH [3]. Among them, collapse is an important marker of the progression of ONFH. Collapse of the articular surface of the femoral head can be secondary to osteoarthritis of the hip, leading to severe hip pain and dysfunction and eventually necessitating joint replacement [4,5]. Therefore, it is important to identify the factors inducing collapse in the early stage of the disease and take appropriate interventions in time for the prognosis of patients.

In recent years, it has been shown that cystic areas can increase the risk of femoral head collapse by disrupting the mechanical stability of the femoral head, thereby accelerating the progression of the disease and affecting the prognosis of patients [6]. The resorption of bone structure disappears and is replaced by tissue as a typical manifestation of Association Research Circulation Osseous (ARCO) stage II, which shows a radiolucent area with reduced density on X-ray [7]. Pathological studies suggest that the cavity in the cystic area is mainly filled by some fibrous granulation like tissue [8]. Femoral head necrosis with cystic areas is more likely to present with microfractures and collapse, suggesting instability of the femoral head structure, so it is believed that cystic areas of femoral head necrosis play an important role in aggravating the process of femoral head collapse [9]. At present, there are two hypotheses about the collapse mechanism of ONFH: one is that osteoclasts are active resulting in reduced bone strength, which reduces their ability to bear the articular surface [10]. The other is that the repaired area is thickened, which produces stress concentration at the junction with the necrotic area [11]. The replacement of the bone in the cystic area by fibrous granulation tissue also forms a cavity inside the femoral head, and the cystic area is mainly located in the weight-bearing area of the femoral head [6]. Most of the previous studies [6,12] have investigated the distribution of cystic degeneration in the femoral head of ONFH patients, and there is a lack of mechanical analysis. The effect of cystic area on femoral head mechanics in patients with ONFH is unknown. We speculated that the cystic area would reduce the weight-bearing ability of the femoral head and destroy the normal mechanical stability of the femoral head, thereby accelerating the collapse of the femoral head. In this study, the incidence and diameter of femoral head collapse and non-collapse cystic degeneration in ONFH patients were retrospectively analyzed, and simulated the stress distribution in the necrotic area and 1 mm area around the cystic area when there were different diameter cystic areas by finite element method. The aim of this study is to investigate the effect of the cystic area on the stress distribution in the femoral head and its role in the progression of the disease and explore the formation mechanism of cystic area of ONFH and its guiding role in the treatment of osteonecrosis.

## 2 Methods

### 2.1 Study subjects

Patients with non-traumatic, non-surgical ONFH admitted to the First Affiliated Hospital of Guangzhou University of Traditional Chinese Medicine from February 2017 to November 2018 were analyzed. Inclusion criteria were: (i) complete imaging data, no femoral head collapse at the first visit (ARCO stage II), (ii) age 20–55 years, and (iii) no history of hip trauma or surgery. Exclusion criteria were: (i) incomplete imaging data during follow-up and (ii) patients with cardiovascular and cerebrovascular diseases or rheumatoid arthritis. The age, gender, etiology, and follow-up time of the patients were recorded, and imaging data such as X-ray and computerized tomography (CT) were collected. All patients were followed up for an average of more than 2 years, and the presence of >2 mm on the surface of the femoral head was defined as collapse [13]. According to whether the femoral head collapsed during the follow-up, the patients were divided into collapse group and non-collapse group. This study was approved by the ethical review board of The First Affiliated Hospital of Guangzhou University of Chinese Medicine (No: Y《2019》 118).

### 2.2 Conservative treatment

All patients accepted the oral administrations with TCM Yuanshi Shengmai Chenggu tablet (six tablets each time, three times per day, institutional approval number: Z20070828) and Fufang Shengmai Chenggu capsule (four capsules each time, three times per day, and institutional approval number: Z20071224). The two drugs were prepared by the First Affiliated Hospital of Guangzhou University of Traditional Chinese Medicine (Guangzhou, China). Yuanshi Shengmai Chenggu tablet is mainly developed from Chinese herbal medicine folium cajani. Fufang Shengmai Chenggu capsule is a formulation of seven herbal medicines, which is prepared by mixing ground forms of folium cajani, angelica sinensis, szechuan lovage rhizome, rehmannia glutinosa, radix paeoniae rubra, crocus sativus, and peach keruel. Following the oral administration, muscle group exercises were performed with an emphasis on anterior flexor muscles, abductor muscles, and adductor muscles and protective weight-bearing exercises. This treatment regimen was approved by the First Affiliated Hospital of Guangzhou University of Traditional Chinese Medicine.

### 2.3 Establishment of 3D models

A 30-year-old healthy woman was excluded from our analysis due to the history of hip and systemic diseases. DICOM data were obtained from CT scans. We constructed a hip joint model through Mimics 16.0 system, Geomagic-Studio 11 system, and Solidworks 2014 software. The Japanese Investigation Committee (JIC) type C1 ONFH was used as a model reference to establish a necrotic zone model. A circle α was drawn around the center of the femoral head using the Solidworks software. Finally, the JIC type C1 ONFH model including the necrotic area was established. Three-dimensional models of ONFH with 0, 5, 10, 15, and 20 mm diameter cystic areas were constructed in the anterolateral weight-bearing area of the femoral head.

### 2.4 Material properties and boundary conditions

**Figure 1:**
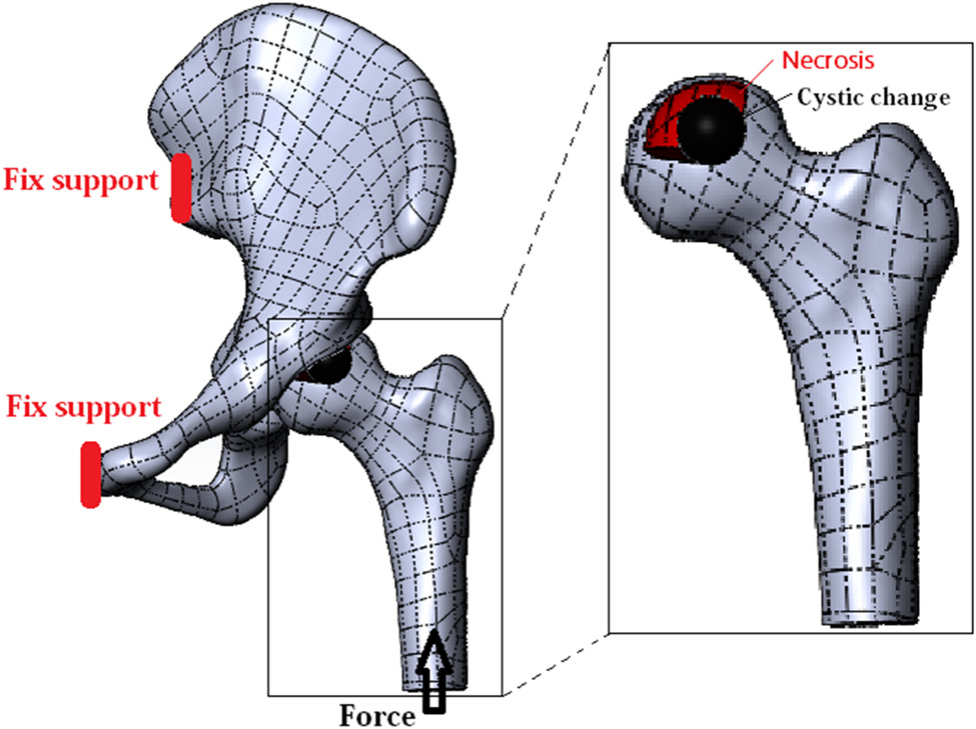
ONFH hip joint model with cystic change area and the diagram of boundary conditions and loading force settings.

The Poisson’s ratio of cystic area was 0.49, and the elastic modulus was 1 MPa [14]. As with details reported by Grecu [15], cortical bone, cancellous bone, and cartilage were defined as isotropic, continuous, and uniform elastic materials. The Poisson’s ratio and elastic modulus of cortical bone, cancellous bone, necrotic tissue, and cartilage are shown in Table 1. The six degrees of freedom at the sacroiliac joint and pubic symphysis were zero. Based on the settings reported by Sverdlova et al. [16], the joint force was 1.6 times the body weight and loaded on the rigid body of the distal femur (Figure 1). Friction was defined as the tangential force between the surfaces of the femoral cartilage and the acetabular cartilage, with a coefficient of friction of 0.2. The binding relation was defined as relationship of other model parts. The results of convergence tests for the element size showed that errors were below 10%.

**Table 1:** Assignment of material properties

Material	Elastic modulus (MPa)	Poisson’s ratio
Cortical bone	15,100	0.3
Cancellous bone	4,457	0.22
Cartilage	10.5	0.45
Necrotic tissue	124.6	0.152
Cystic lesion	1	0.49

### 2.5 Observation indicators

In terms of clinical studies, age, gender, etiology, height, weight, BMI, JIC classification, presence of cystic areas, and diameter of cystic areas were compared between the collapse and non-collapse groups. The bone structure disappeared, and the main manifestation was hypodense radiolucent area on CT images, according to which it was determined whether the affected hip contained cystic area. On the CT image of femoral head, take the middle position of coronal plane of cystic area, measure the size of their mutually perpendicular long diameter and transverse diameter, and take the average value of long diameter and transverse diameter as the diameter of cystic area. In addition, record the maximum von Mises stress and the average von Mises stress on the head surface (take the corresponding 10-point von Mises stress on the head surface of different models and calculate its average). Furthermore, the maximum von Mises stress and mean von Mises stress in the necrotic area were measured to analyze the relationship between the two and the diameter of the cystic area. Finally, the maximum von Mises stress in the 1 mm area around the cystic area was measured: the effect of the cystic area on the stress distribution in the surrounding area was analyzed, and a spherical shell with a radial extension of 1 mm around the simulated cystic area was used as the area to measure the von Mises stress [17]. The relationship between the maximum stress in this region and the diameter of the cystic region was analyzed.

### 2.6 Statistical methods

SPSS 24.0 statistical software was used for analysis. Measurement data were expressed as mean ± standard deviation, independent sample *t*-test was used for comparison, enumeration data were expressed as rate, and *χ* ^2^ test or rank sum test was used for comparison. Simple linear regression analysis was used to analyze the relationship between the diameter of the bone resorption area and the maximum von Mises stress in the cortical bone of the femoral head, the maximum von Mises stress in the necrotic area, and the maximum von Mises stress in the 1 mm area around the cysts. *P* < 0.05 was considered statistically significant.


**Ethical approval:** This study was conducted in agreement with the Declaration of Helsinki and its later amendments or comparable ethical standards and had been approved by the ethics board of The First Affiliated Hospital of Guangzhou University of Chinese Medicine (No: Y《2019》 118). All participants provided informed consent before their participation in the study and written consent was obtained from all participants.

## 3 Results

### 3.1 General information

A total of 85 patients (44 males and 41 females) with 106 hips, including 21 patients with ONFH, were enrolled in this study. The age ranged from 21 to 60 years with a mean age of 37.4 ± 8.12 years. The follow-up time was 2–4 years, with an average of 2.8 ± 0.6 years. Forty-five patients (57 hips) with ONFH who had femoral head collapse were included in the collapse group, and the remaining 40 patients (49 hips) were included in the non-collapse group. There was significant difference in JIC classification between the two groups (*P* < 0.05), most of which were C2 type in the collapse group and B type in the non-collapse group. There was a difference between the two groups on whether there was a cystic area in the femoral head, with 49.1% in the collapse group showing a cystic area, which was significantly higher than that in the non-collapse group (18.4%), and the difference was statistically significant (*P* < 0.05). In addition, the diameter of the cystic areas was significantly larger in the collapsed group than in the non-collapsed group (*P* < 0.05). There was no significant difference in age, gender, height, weight, BMI, and etiology between the two groups (*P* > 0.05) (Table 2).

**Table 2:** Comparison of general data between the two groups

Parameters	Collapse group (*n* = 45 [57 hips])	Non-collapse group (*n* = 40 [49 hips])	*t*/*χ* ^2^	*P*-value
Age (years)	37.0 ± 11.36	37.9 ± 6.70	–0.496	0.621
Gender, *n* (%)				
Male	23(51.1%)	21(52.5%)	0.016	0.898
Female	22(48.9%)	19(47.5%)		
Height (cm)	164.6 ± 7.43	164.7 ± 7.26	–0.047	0.962
Weight (kg)	65.7 ± 13.6	63.9 ± 11.6	0.731	0.466
BMI (kg/m^2^)	23.4 ± 3.68	23.8 ± 3.97	0.501	0.617
Etiology, *n* (%)				
Steroid	16(35.6%)	14(35.0%)	0.008	0.996
Alcoholic	17(37.8%)	15(37.5%)		
Idiopathic	12(26.7%)	11(27.5%)		
JIC type, *n* (%)				
Type-A	0(0%)	2(5.0%)	37.25	0.000
Type-B	6(13.3%)	28(70.0%)		
Type-C1	18(40.0%)	9(22.5%)		
Type-C2	21(46.7%)	1(2.5%)		
Cystic change area, *n* (%)				
Yes	28(49.1%)	9(18.4%)	10.969	0.0012
No	29(50.9%)	40(81.6%)		
Cystic change area diameter (mm)	15.8 ± 5.33	6.3 ± 2.94	10.995	0.000

### 3.2 Von Mises stress values for different capsule diameter models

**Figure 2:**
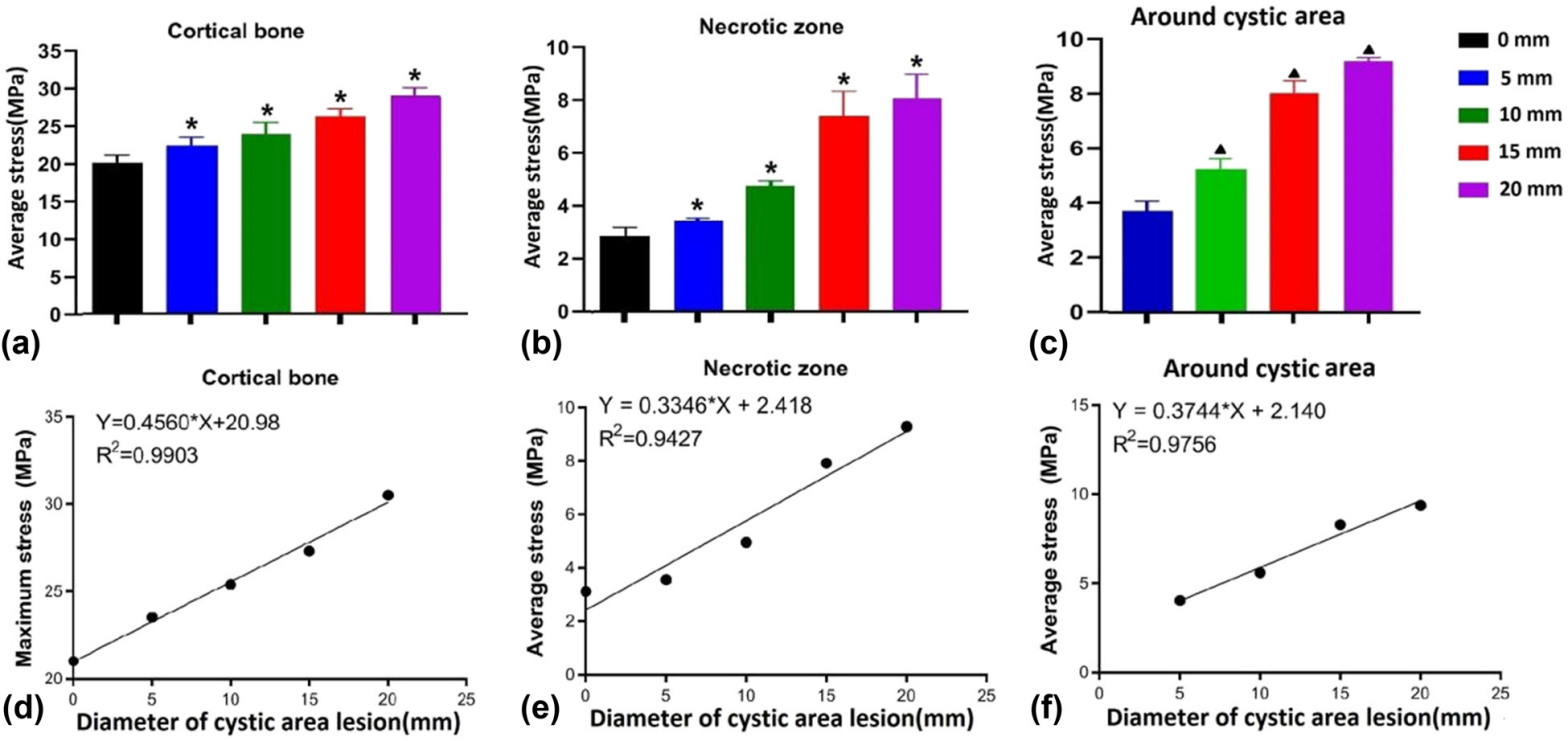
The average stress of each model and relationship between maximum stress and cystic area diameter: (a–c) different models average stress of cortical bone, necrotic zone, and around cystic area, (d–f) relationship between the cortical bone, necrotic zone, and around cystic area maximum von Mises stress of model and cystic area diameter. *: Compared with the 0 mm model, the difference was statistically significant. ▲: Compared with the 5 mm model, the difference was statistically significant.

The maximum and average stress values of cortical bone in the weight-bearing area of femoral head were the smallest in the 0 mm diameter cystic area model. The mean stress value of cortical bone in the weight-bearing area of the femoral head increased with the increase in the diameter of the resorption area, and the difference was statistically significant (*P* < 0.05). Similarly, the 0 mm model had the smallest mean and maximum stresses in the necrotic zone. The mean stress value of the necrotic area increased with the increase in the diameter of the absorption area, and there was no statistically significant difference between the 15 mm model and the 20 mm model, and the other differences were statistically significant (*P* < 0.05). Furthermore, the maximum stress and mean stress around the cystic area increased with the increase in the diameter of the absorption area, and there was a significant difference in the mean stress around the cystic area (*P* < 0.05) (Table 3 and Figure 2).

**Table 3:** Von Mises stress values (MPa) for different capsule diameter models

Grouping	Maximum stress of cortical bone	Average stress of cortical bone	Maximum stress of necrotic area	Average stress of necrotic area	Maximum stress of cystic area	Average stress of cystic area
0 mm	21	20.19 ± 1.01	3.11	2.85 ± 0.33	—	—
5 mm	23.5	22.45 ± 1.13*	3.55	3.43 ± 0.09*	4.03	3.72 ± 0.36
10 mm	25.4	24.0 ± 1.52*	4.95	4.75 ± 0.17*^#^	5.59	5.23 ± 0.38^#^
15 mm	27.3	26.3 ± 1.07*^#▲^	7.92	7.40 ± 0.94*^#▲^	8.29	8.01 ± 0.46^#▲^
20 mm	30.5	29.1 ± 1.10*^#▲★^	9.29	8.08 ± 0.90*^#▲^	9.37	9.19 ± 0.13^#▲★^
*F*-value	—	84.35	—	147.74	—	1416.9
*P*-value	—	<0.001	—	<0.001	—	<0.001

*: Compared with the 0 mm model, the difference was statistically significant. #: Compared with the 5 mm model, the difference was statistically significant. ▲: Compared with the 10 mm model, the difference was statistically significant. ★ Compared with the 15 mm model, the difference was statistically significant.

### 3.3 Relationship between the von Mises stress of model and cystic area diameter

The maximum von Mises stresses in the femoral head cortex were 21, 23.5, 25.4, 27.3, and 30.5 in the 0, 5, 10, 15, and 20 mm finite element models of the cystic region diameter, respectively. The relationship between the diameter of the cystic area and the maximum von Mises stress of the femoral head cortical bone was fitted by a linear regression curve, and the results showed that the diameter of the cystic area was linearly positively correlated with the maximum von Mises stress of the femoral head cortical bone (*R* ^2^ = 0.9903, *F* = 307.0, *P* = 0.0004), and the fitted regression equation was: *Y* = 0.4560 * *X* + 20.98. In addition, the maximum von Mises stresses in the necrotic zones were 3.11, 3.55, 4.95, 7.92, and 9.29 in the finite element models with 0, 5, 10, 15, and 20 mm capsule diameter, respectively. The relationship between the diameter of the cystic area and the maximum von Mises stress in the necrotic area was fitted by a linear regression curve, and the results showed that the diameter of the cystic area was linearly positively correlated with the maximum von Mises stress in the necrotic area (*R* ^2^ = 0.9427, *F* = 49.40, *P* = 0.0059), and the fitted regression equation was: *Y* = 0.3346 * *X* + 2.418 (Figure 2).

### 3.4 Relationship between von Mises stress around the cystic area and the diameter of the cystic area

**Figure 3:**
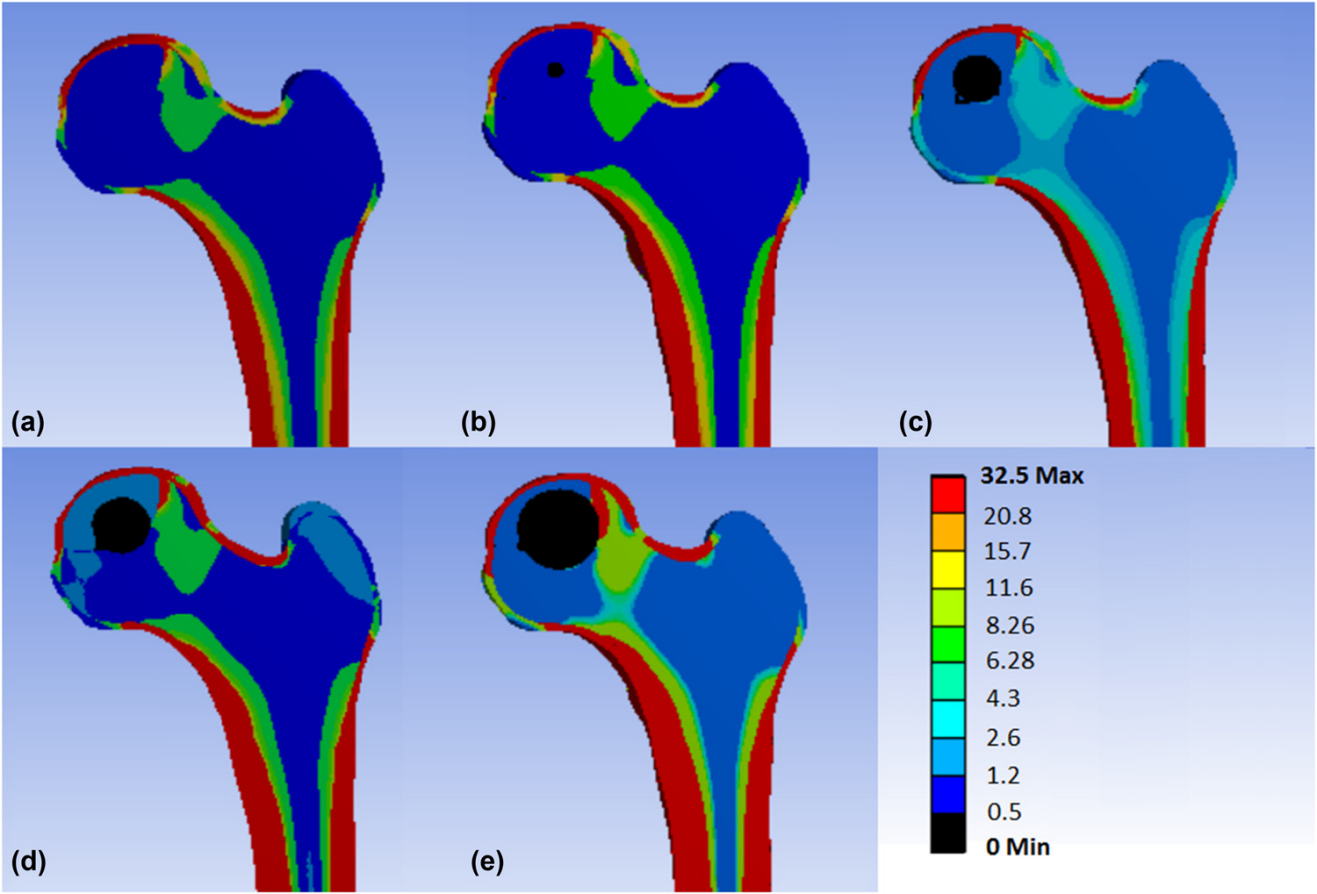
Finite element stress cloud diagrams of different models of femur: (a) diameter of cystic change area 0 mm, (b) diameter of cystic change area 5 mm, (c) diameter of cystic change area 10 mm, (d) diameter of cystic change area 15 mm, and (e) diameter of cystic change area 20 mm.

The maximum von Mises stresses around the cystic area were 4.03, 5.59, 8.29, and 9.37 in the 5, 10, 15, and 20 mm diameter finite element models of the cystic area, respectively. The relationship between the diameter of the cystic area and the maximum von Mises stress around the cystic area was fitted by linear regression curve. The results showed that the diameter of the cystic area was linearly positively correlated with the maximum von Mises stress of the cortical bone of the femoral head (*R* ^2^ = 0.9756, *F* = 79.92, *P* = 0.0123), and the fitted regression equation was *Y* = 0.4560 * *X* + 20.98 (Figure 2). The finite element stress distribution clouds show that there is a stress concentration area above the cystic region, and this region range gradually increases with the increase in the diameter of the cystic region (Figure 3).

### 3.5 Typical case

**Figure 4:**
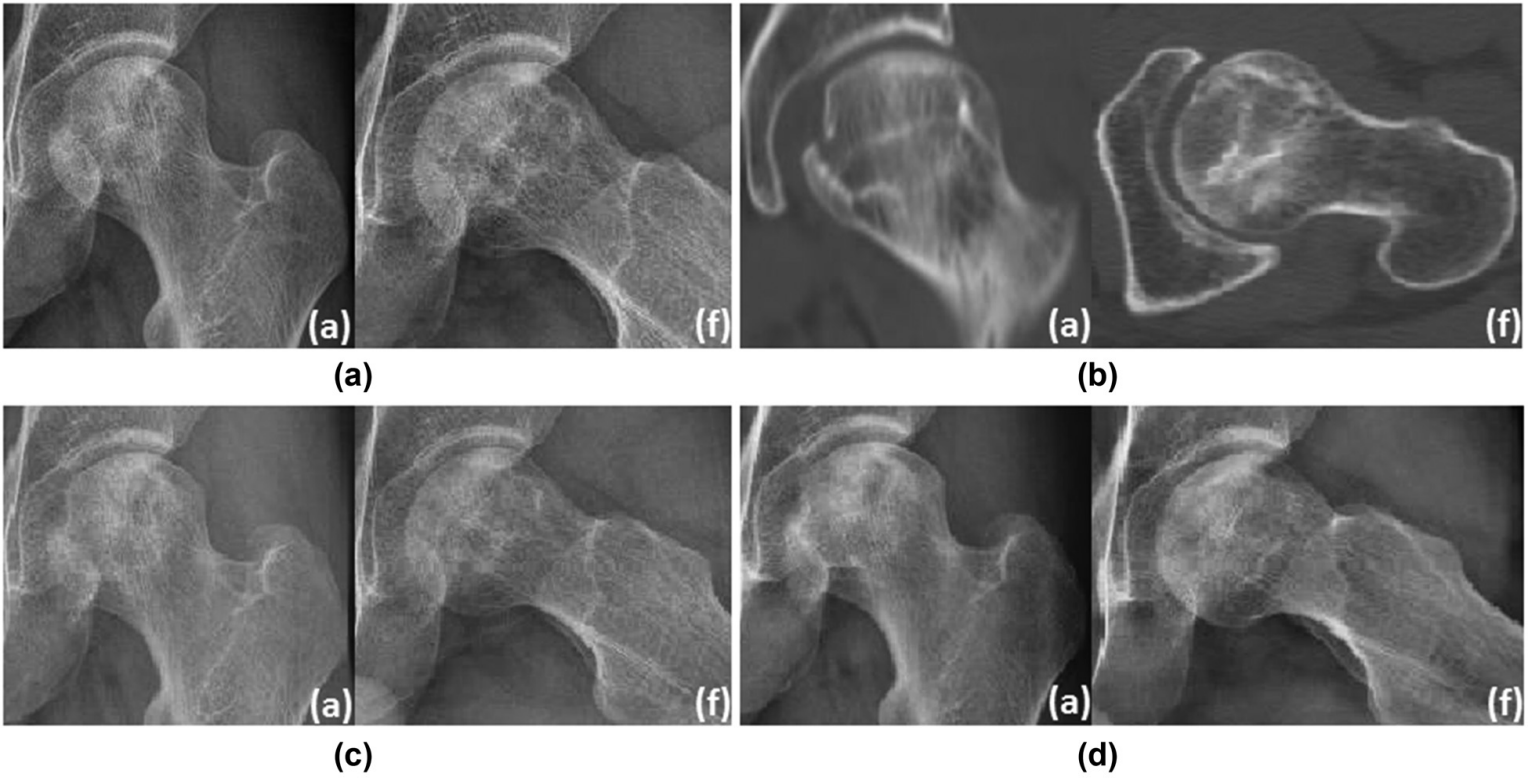
Typical case of ONFH without cystic area: (a) (radiograph imaging at an anteroposterior (a) and frog (f) lateral view indicated extensive necrosis of the left femoral head), (b) CT showed no obvious cystic change area, (c) 6 months follow-up, and (d) 1.5 years follow-up.

**Typical case 1**: Male, 36 years, with left hip pain for more than 7 months, diagnosed as steroid-induced ONFH. This is an ONFH patient with ARCO II and JIC type B. CT showed no obvious cystic change area. After 6 months of non-surgical hip preservation treatment, the area of necrosis was reduced and the joint space was acceptable. After 1.5 years of non-surgical hip preservation treatment, the femoral head did not collapse significantly, and the density of the necrotic area increased (Figure 4).

**Figure 5:**
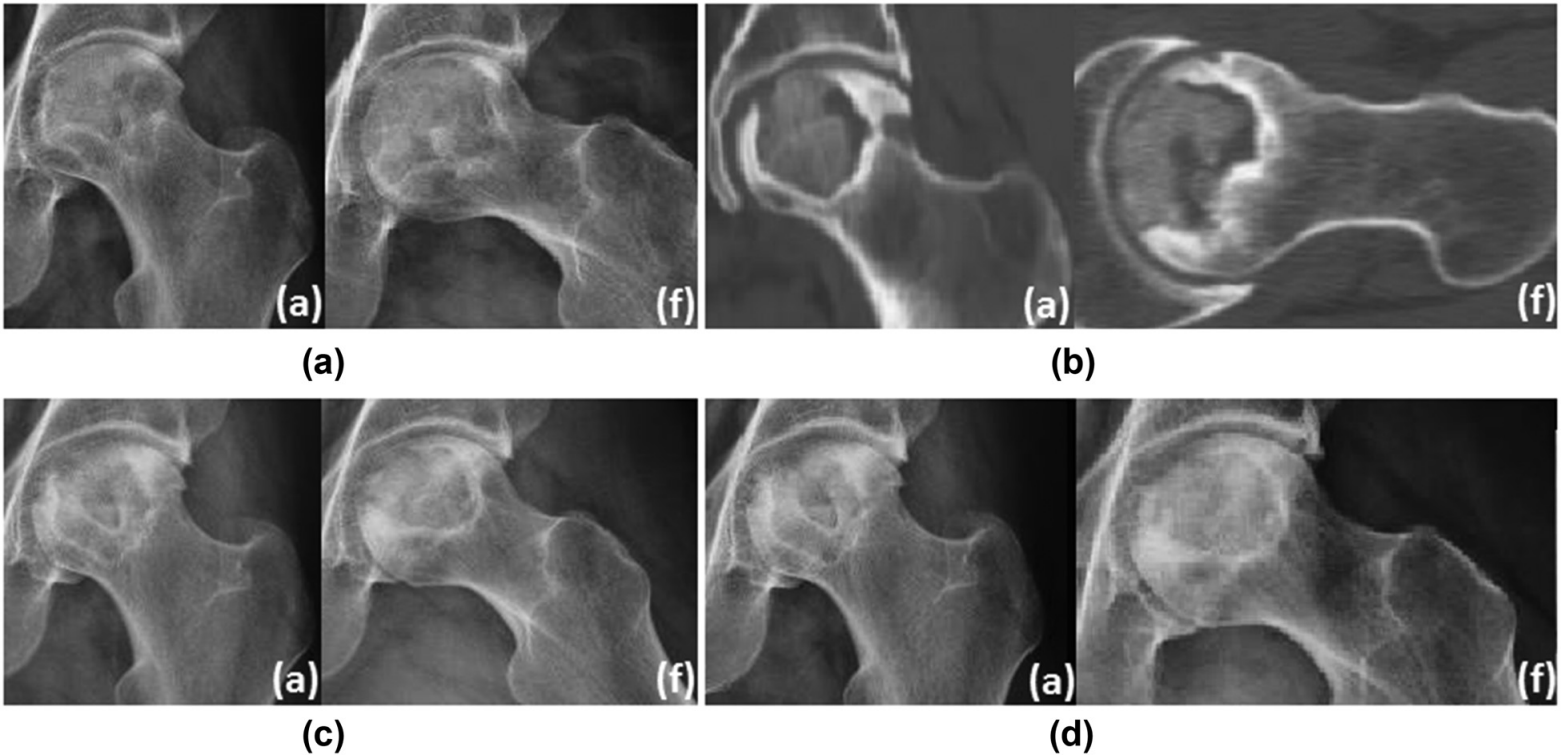
Typical case of ONFH with cystic area: (a) (radiograph imaging at an anteroposterior (a) and frog (f) lateral view showed large-scale necrosis of the left femoral head), (b) CT showed obvious cystic area, (c) 1 year follow-up, and (d) 2 years follow-up.

**Typical case 2:** Male, 45 years, with left hip pain for more than 9 months, diagnosed as steroid-induced ONFH graded as JIC type B. CT showed obvious cystic change area. After 1 year of non-surgical hip-preserving treatment, the femoral head collapsed and the patient’s pain became a little worse. After 2 years of non-surgical hip preservation treatment, the femoral head has become slightly flat (Figure 5).

## 4 Discussion

The cystic area is a cavity formed in the femoral head, and the cystic area is mainly located in the weight-bearing area of the femoral head. However, the effect of cystic areas on femoral head mechanics in patients with ONFH is unknown. Therefore, we carried out a clinical retrospective study with finite element analysis. Our clinical study found cystic areas in 49.1% of patients in the collapse group, which was significantly higher than that in the non-collapse group (18.4%). In addition, the diameter of the cystic areas was significantly larger in the collapsed group than in the non-collapsed group. The finite element results showed that the maximum stress and average stress in the necrotic area increase gradually with the increase in the diameter of the cystic area, and there was a stress concentration area around the cystic area, and the maximum stress around it also increased with the increase in the diameter of the cystic area.

The prognosis of ONFH mainly depends on whether the articular surface is collapsed [18,19]. The disappearance of bone structure inside the cystic area can further reduce the weight-bearing ability of the femoral head and increase the risk of femoral head collapse, thereby accelerating disease progression [11]. The location of the lesion in the necrotic area is considered to be an important factor in collapse. One study found that the prognosis is often very poor when the necrotic area involves the lateral weight-bearing area [20]. Kubo et al. [21] found that femoral head necrosis had a high collapse rate even when the necrotic focus involved more anterior regions, even when it was medial. The cystic area is mainly located in the anterolateral aspect of the femoral head, which may further reduce the mechanical strength and weight-bearing capacity of the femoral head and accelerate collapse. The sclerotic zone can delay or prevent collapse of the femoral head by providing mechanical support to the femoral head [22]. However, most of the cystic areas were connected to the sclerotic zone, which destroyed the integrity of the sclerotic margin, thereby reducing the protection of necrotic tissue and further increasing the risk of femoral head collapse [23]. The researchers analyzed the location characteristics of the cystic areas in the CT images and found that the cystic areas were mainly located in the intermediate and lateral columns of the femoral head. They concluded that this compromises the integrity of the principal compressive stress in the femoral head, damages the normal stress transfer pathway in the femoral head, increases the average stress in the femoral head, and thus accelerates the collapse of the femoral head [6]. In this study, we found that patients with ONFH had an increased risk of collapse when cystic areas appeared in the femoral head. In addition, the diameter of cystic areas was larger in the collapsed group than in the non-collapsed group. The reason for the differential formation of cystic areas remains unclear. Durr et al. [24] suggest that stress-induced microfracture may be the first step in the development of subchondral bone cysts. Secondary effects, such as osteoclast resorption, then induce the cystic lesion itself. Moreover, another study [6] suggested that cystic lesions are related to osteoclastic resorption of necrotic trabeculae with fibrous replacement of bone and the bone resorption of osteoclasts is due to excessive stress.

The data of CT yielded that the cystic areas were mainly located on the anterior, intermediate, lateral columns of the femoral head, and near the sclerotic zone [6]. Therefore, it is speculated that the formation of cystic areas may be due to stress-induced bone resorption. At stress concentrations in the interior of the femoral head, bone microfractures occur when the stress on the bone exceeds its maximum bearing strength [25]. Constant stress action or increased weight-bearing can allow this process to occur repeatedly, with subsequent resorption of fracture fragments by osteoclasts and replacement by fibrous granulation tissue [26]. It has been reported in the literature that during the occurrence and development of osteoarthritic cystic areas, pathological changes caused by increased stress can enhance macrophage responses, and macrophages promote osteoclast resorption in the subchondral bone around osteoarthritic cystic areas through macrophage-osteoclast differentiation and play a role in the osteolysis process of increased cystic areas. Because similar tissue components exist between osteoarthritis and cystic areas of ONFH, biological factors may also help to promote the maintenance and expansion of cystic areas in ONFH [27]. However, at present we have not further tested the hypothesis. In this study, according to the results of the previous studies, the main part of the simulated cystic area was designed to be located in the anterolateral region of the femoral head, which was in line with the actual distribution characteristics of the cystic area in clinical practice. Previous studies have confirmed that the reduction of bone structure strength in the necrotic area and the appearance of stress concentration areas in the femoral head are the main causes of collapse [28]. The results of our finite element study suggest that the formation of the cystic area increases the maximum stress and mean stress of the necrotic area, and there is a stress concentration area around the cystic area, thus increasing the risk of bone fracture. Although the optimal treatment strategy for ONFH remains controversial, it is generally believed that aggressive hip preservation treatment before collapse can improve the prognosis [29,30].Considering that cystic areas increase the risk of collapse, we recommend that patients with cystic areas should be carefully evaluated and closely followed up in order to take timely intervention measures when the disease progresses.

The innovation of this study was to investigate the relationship between cystic areas and femoral head collapse, which was verified by finite element analysis. However, there are still some limitations in this study. First, the clinical part of this study is a single-center retrospective analysis study with a limited number of cases, which may have selection bias. Secondly, our study only investigated the effect of areas with a high incidence of cystic areas on the mechanical properties of the femoral head, and the observation of the morphological characteristics of cystic areas also only set up an ideal model of spherical cystic areas. In fact, the shape of cystic areas is irregular, some cystic areas are surrounded by sclerotic areas, and the cystic areas located in the anterolateral side may also destroy the subchondral bone plate, so it is necessary to further analyze the effect of other areas and cystic areas with different morphological characteristics on the progression of ONFH.

## 5 Conclusion

The maximum stress and average stress of necrotic area can be increased in cystic area, and the increase of stress in cystic area is more obvious. Stress concentration areas can be generated around the cystic areas. The presence and increased diameter of the cystic areas accelerates the collapse of the ONFH femoral head.
