# Erratum to “Effect of femoral head necrosis cystic area on femoral head collapse and stress distribution in femoral head: A clinical and finite element study”

**DOI:** 10.1515/med-2022-0563

**Published:** 2022-09-20

**Authors:** Tianye Lin, Zhaoming Zhang, Yuan Zhong, Wenting Song, Peng Yang, Ding Wang, Fan Yang, Qingwen Zhang, Qiushi Wei, Wei He

**Affiliations:** Department of Orthopedics, The First Clinical Medical College of Guangzhou University of Chinese Medicine, Guangzhou, Guangdong 510405, China; Department of Orthopedics, Affiliated Foshan Hospital, Guangzhou University of Traditional Chinese Medicine, Foshan 528000, China; Department of Orthopedics, Guangdong Research Institute for Orthopedic & Traumatology of Chinese Medicine, Guangzhou, Guangdong 510240, China; Department of Joint Orthopaedic, The Third Affiliated Hospital, Guangzhou University of Chinese Medicine, Guangzhou, Guangdong 510240, China

In the published article Zhang Z, Lin T, Zhong Y, Song W, Yang P, Wang D, Yang F, Zhang Q, Wei Q, He W. Effect of femoral head necrosis cystic area on femoral head collapse and stress distribution in femoral head: A clinical and finite element study. Open Med. (Wars) 2022 Jul 13;17(1):1282–91. doi: 10.1515/med-2022-0506. PMID: 35892078; PMCID: PMC9281584, authors requested to change the order of the co-authors, making Dr Tianye Lin as the first author. As a result of that the correct order of the authors is as follows:

Tianye Lin, Zhaoming Zhang, Yuan Zhong, Wenting Song, Peng Yang, Ding Wang, Fan Yang, Qingwen Zhang, Qiushi Wei and Wei He.

